# Development and Validation of the Nurse Unit Manager Competency Scale (NUM‐CS): A Multicenter Study

**DOI:** 10.1155/jonm/9388493

**Published:** 2026-08-04

**Authors:** Jianmei Chen, Zhengwei Lan, Shuxia Xie, Jianwen Li, Nianhao Zhong, Xiaoyan Tan, Haixin Jiang, Minjuan Zhou

**Affiliations:** ^1^ Department of Emergency Nursing, Guangzhou Chest Hospital Affiliated to Guangdong Pharmaceutical University, Guangzhou 510095, China; ^2^ Department of Nursing, Guangzhou Chest Hospital Affiliated to Guangdong Pharmaceutical University, Guangzhou 510095, China; ^3^ Department of Drug-Resistant Tuberculosis, Guangzhou Chest Hospital Affiliated to Guangdong Pharmaceutical University, Guangzhou 510095, China; ^4^ Department of Tuberculosis Interventional, Guangzhou Chest Hospital Affiliated to Guangdong Pharmaceutical University, Guangzhou 510095, China; ^5^ Department of Infectious Diseases and Immunology, Guangzhou Chest Hospital Affiliated to Guangdong Pharmaceutical University, Guangzhou 510095, China; ^6^ Department of Tuberculosis Comorbidity, Guangzhou Chest Hospital Affiliated to Guangdong Pharmaceutical University, Guangzhou 510095, China

**Keywords:** competency scale, Nurse Unit Manager, nursing management, psychometric validation, situational judgment test

## Abstract

**Aim:**

To develop and validate the Nurse Unit Manager Competency Scale (NUM‐CS) and to examine the stability of its factor structure across hospital settings in China.

**Background:**

Nurse Unit Managers (NUMs) play a central role in clinical governance and care delivery. In China, NUM appointments are commonly based on seniority rather than formal competency assessment. Existing international frameworks provide useful reference points but do not fully reflect the demands of specialized and high‐risk hospital contexts. A context‐sensitive and psychometrically robust instrument is, therefore, needed to support competency‐based nursing management.

**Methods:**

A multiphase methodological study was conducted between May and December 2025. Items were generated from a prior qualitative study and refined through two rounds of Delphi consultation. Psychometric testing was performed using two independent samples: a specialized hospital sample (*n* = 151) for exploratory factor analysis (EFA) and a cross‐setting sample (*n* = 212) for confirmatory factor (CFA) analysis. Reliability, construct validity, and criterion‐related validity were examined.

**Results:**

The final NUM‐CS consisted of 33 self‐report items across six competency domains. EFA showed excellent sampling adequacy (KMO = 0.953; Bartlett’s *χ* ^2^ = 5248.363, *p* < 0.001), accounting for 74.28% of the total variance. CFA supported the six‐factor model across hospital settings. Internal consistency was high across all domains. Total NUM‐CS scores were positively correlated with the 12‐item situational judgment test score (*r* = 0.312, *p* < 0.001).

**Conclusion:**

The NUM‐CS is a preliminarily validated, practice‐oriented tool for identifying and developing NUM reserve candidates across diverse hospital settings in China.

**Implications for Nursing Management:**

The NUM‐CS offers a structured tool to support competency‐based identification, development, and formative assessment of NUM reserve candidates, particularly in specialized and high‐risk clinical environments.

## 1. Introduction

Nurse Unit Managers (NUMs) are responsible for coordinating unit‐level clinical operations, managing nursing staff and resources, and ensuring the delivery of safe and effective patient care within increasingly complex healthcare systems [1]. Leadership and management competencies of nurse managers are widely recognized as essential determinants of effective nursing management and have been associated with improved practice environments, reduced missed nursing care, and better patient outcomes [2].

Despite the acknowledged importance of managerial and leadership competencies, the conceptualization and measurement of nurse manager competence remain inconsistent. International literature has identified multiple competency domains relevant to NUMs; however, definitions vary considerably, and existing assessment approaches lack standardization. A recent scoping review of head nurse competency instruments similarly highlighted inconsistencies in terminology, competency descriptions, and measurement approaches, indicating that clearer and more standardized competency definitions are still needed for practice‐based assessment [3]. To date, no universally accepted instrument comprehensively captures NUM competencies across diverse healthcare settings [4, 5].

Moreover, instruments developed to assess nurse manager competencies differ substantially in structure, content, and psychometric quality. A recent systematic review identified more than ten competency‐related tools, with varying emphasis on managerial, clinical, or specialized competencies, many of which lacked rigorous evaluation of measurement properties or evidence of cross‐context applicability [4]. Recent psychometric validation studies have also supported the multidimensional nature of nurse manager competencies, while suggesting that further validation across healthcare systems and institutional contexts remains necessary [6, 7]. In China, although competency frameworks and validated instruments have been developed for specific nursing roles—such as research nurses and clinical nurse specialists—comprehensive and psychometrically robust tools specifically designed for NUMs remain scarce [8].

The conceptual framework of the Nurse Unit Manager Competency Scale (NUM‐CS) was informed by three complementary theoretical perspectives: a competency‐based role perspective, nursing leadership competency frameworks, and clinical governance. Competency‐based role theory emphasizes observable knowledge, skills, behaviors, motives, and role‐related capabilities required for effective performance [9, 10]. Nursing leadership competency frameworks highlight the leadership, communication, personnel management, professional development, and organizational coordination capabilities required of nurse managers [4, 5, 11, 12]. Clinical governance further emphasizes accountability for quality improvement, patient safety, risk management, and evidence‐informed practice at the unit level [1, 13]. Together, these perspectives informed the six‐domain structure of the NUM‐CS and guided item generation, Delphi consultation, and psychometric validation.

This study aimed to develop and preliminarily validate a competency‐based assessment instrument for identifying and developing NUM reserve candidates across hospital settings in China.

## 2. Methods

### 2.1. Study Design and Overview

This multiphase methodological study was conducted to develop and psychometrically validate the NUM‐CS. The scale development followed established guidelines for instrument development and comprised four sequential phases: (1) item generation based on a prior qualitative study of NUM competencies in specialized hospital settings; (2) content validation through a two‐round Delphi expert consultation; (3) pilot testing and item refinement; and (4) large‐sample psychometric evaluation, including exploratory factor analysis (EFA), confirmatory factor analysis (CFA), reliability testing, and criterion‐related validity assessment. The NUM‐CS was developed as a self‐report competency scale, whereas the situational judgment test (SJT) was used only as an external criterion measure for examining criterion‐related validity and was not included as part of the scale structure. Reporting of this study followed the STROBE guidelines, and the completed checklist is provided as supporting information.

Two independent samples were used to strengthen the robustness of the scale structure. A homogeneous specialized hospital sample was used for EFA, while a separate cross‐setting sample drawn from diverse hospital types was used for CFA to evaluate the stability of the proposed competency framework across institutional contexts.

### 2.2. Item Generation

Initial items for the NUM‐CS were generated based on findings from a prior qualitative study exploring the competencies required of NUMs in specialized hospital settings. That qualitative phase employed a grounded theory approach and involved in‐depth interviews and focus group discussions with NUMs and key nursing stakeholders. The analysis identified core competency domains reflecting the complex managerial, clinical governance, and leadership demands faced by NUMs in high‐risk and specialized healthcare environments.

Based on the qualitative themes and subthemes, an initial pool of items was developed using behavioral statements to reflect observable managerial practices rather than abstract traits. Item wording was informed by participants’ original expressions where appropriate and was designed to capture typical behaviors exhibited by competent NUMs in daily practice. Each item was formulated to assess a single competency dimension, avoiding double‐barreled statements, ambiguous wording, and technical jargon.

The initial self‐report item pool consisted of 35 competency items covering six preliminary competency domains: communication and cross‐boundary coordination, leadership and personnel management, clinical governance and risk management, decision‐making and problem‐solving, team development and talent cultivation, and professional self‐regulation. These self‐report items were rated on a five‐point Likert scale ranging from 1 (“*strongly disagree*”) to 5 (“*strongly agree*”), with higher scores indicating higher levels of perceived competency. In parallel, a scenario‐based SJT module was developed as an external criterion measure of applied managerial judgment and was not included in the NUM‐CS scale structure. This preliminary version of the self‐report scale was subsequently subjected to expert content validation through a Delphi process.

### 2.3. Content Validity (Delphi Study)

Content validity of the preliminary NUM‐CS was evaluated using a Delphi expert consultation process. A total of 15 nursing experts were purposively recruited based on predefined criteria, including a senior professional title (associate chief nurse or above), extensive experience in nursing management, and familiarity with nurse unit management in specialized hospital contexts. All 15 experts completed the Delphi ratings independently and anonymously. No group discussion or feedback exchange occurred between experts across or within rounds. The expert panel represented multiple hospitals and regions, ensuring diversity of professional perspectives.

The Delphi consultation consisted of two consecutive rounds conducted between October and November 2025. The first round was carried out from 8 October to 22 October 2025, and the second round from 3 November to 12 November 2025. In each round, experts independently rated each item for relevance and clarity using a four‐point Likert scale (1 = *not relevant/unclear* and 4 = *highly relevant/very clear*). Open‐ended comment fields were provided to allow experts to suggest modifications, deletions, or additions to the items.

Item‐level content validity index (I‐CVI) and scale‐level content validity index (S‐CVI/Ave) were calculated to quantify content validity. An I‐CVI value of ≥ 0.78 was considered acceptable for item retention, while an S‐CVI/Ave value of ≥ 0.90 indicated satisfactory overall content validity. Kendall’s coefficient of concordance (W) was used to assess the degree of agreement among experts, with statistical significance indicating acceptable consensus.

Following the first Delphi round, items that failed to meet the predefined I‐CVI threshold or were identified by experts as unclear or redundant were revised or removed based on both quantitative indices and qualitative feedback. The revised item pool was subsequently redistributed to the same panel of experts for the second Delphi round. After two rounds of consultation, expert consensus was achieved on item relevance and clarity, resulting in a refined version of the NUM‐CS with satisfactory content validity.

### 2.4. Participants and Sampling

A stratified sampling strategy was used to recruit participants from multiple hospitals across China. Two independent samples were collected for different stages of psychometric evaluation.

#### 2.4.1. Specialized Hospital Sample (EFA)

The EFA sample was recruited from 15 hospitals of the same category, primarily chest hospitals, tuberculosis hospitals, infectious disease hospitals, and public health–oriented institutions. These hospitals were distributed across multiple regions in China and shared similar clinical characteristics, including high patient acuity and intensive infection prevention and control requirements.

A total of 151 eligible nurses from these specialized hospitals were included in the final EFA sample.

#### 2.4.2. Cross‐Setting Hospital Sample (CFA)

The CFA sample was drawn from 55 hospitals across different categories, including large tertiary general hospitals, specialized hospitals, traditional Chinese medicine hospitals, maternal and child health institutions, and regional people’s hospitals. These hospitals represented diverse organizational contexts and levels of service complexity across different regions of China.

In total, 212 eligible nurses were included in the cross‐setting sample used for CFA.

#### 2.4.3. Inclusion and Exclusion Criteria

Participants were eligible for inclusion if they met the following criteria:

1. Held a professional title of charge nurse or above and
2. Were under 50 years of age, to ensure realistic eligibility for future NUM appointments given fixed‐term roles and statutory retirement around age 55 in China.

Nurses who were currently serving as NUMs (head nurses) were excluded.

### 2.5. Data Collection Procedures

Data collection was conducted between May and December 2025. All data were collected using an online survey platform (Wenjuanxing). Potential participants were invited to complete the questionnaire through study links distributed via WeChat or email. For each participating hospital, a designated local contact person was responsible for disseminating the survey link to nurses who met the inclusion criteria.

Prior to accessing the questionnaire, participants received written information describing the study purpose, the voluntary nature of participation, and the confidentiality of responses. The questionnaire was completed anonymously, and submission of the questionnaire was considered to indicate informed consent. No identifying personal information was collected, and all responses were used solely for research purposes.

Returned questionnaires were screened for completeness and validity prior to analysis. Questionnaires with substantial missing data or clearly patterned or inconsistent response patterns were excluded from further analysis. All valid questionnaires were exported from the survey platform and entered into a secure database for subsequent statistical analysis.

### 2.6. Psychometric Evaluation

Psychometric evaluation of the NUM‐CS included item analysis, EFA, CFA, reliability assessment, and criterion‐related validity testing. EFA was conducted in the specialized hospital sample to explore the latent structure of the self‐report items, whereas CFA was conducted in the independent cross‐setting sample to test the theoretically proposed competency structure and compare alternative measurement models.

#### 2.6.1. Item Analysis

Item performance was examined using descriptive statistics and corrected item–total correlations (CITCs). Items with low item–total correlations or poor contribution to internal consistency were considered for removal. Internal consistency was assessed using Cronbach’s alpha coefficient, with values of ≥ 0.70 considered acceptable.

#### 2.6.2. EFA

EFA was performed using the specialized hospital sample to examine the underlying factor structure of the self‐report NUM‐CS items. Prior to factor extraction, data suitability was assessed using the Kaiser–Meyer–Olkin (KMO) measure of sampling adequacy and Bartlett’s test of sphericity. Factors were extracted using principal axis factoring, with oblique rotation applied to allow for correlations among factors. Decisions regarding factor retention were based on eigenvalues greater than 1.0, inspection of the scree plot, factor loadings, cross‐loadings, and conceptual interpretability.

#### 2.6.3. CFA

CFA was conducted using the cross‐setting hospital sample to test the theoretically proposed factor structure and compare alternative measurement models. Model fit was evaluated using multiple goodness‐of‐fit indices, including the chi‐square statistic and its ratio to degrees of freedom (χ^2^/df), the comparative fit index (CFI), the Tucker–Lewis index (TLI), and the root mean square error of approximation (RMSEA). Conventional criteria were used to judge acceptable model fit.

Composite reliability (CR) and average variance extracted (AVE) were calculated from the standardized factor loadings of the correlated six‐factor CFA model to assess CR and convergent validity. Discriminant validity was examined using inter‐factor correlations and the Heterotrait–Monotrait (HTMT) ratio of correlations, which was calculated from the interitem correlation matrix of the independent CFA sample. CR values ≥ 0.70, AVE values ≥ 0.50, and HTMT values < 0.90 were considered acceptable.

#### 2.6.4. Reliability Assessment

Internal consistency reliability of the overall scale and each subscale was assessed using Cronbach’s alpha coefficients. Where applicable, CR was also examined to further evaluate the stability of the scale structure.

#### 2.6.5. Criterion‐Related Validity

Criterion‐related validity was examined using a brief SJT module designed to assess managerial decision‐making in typical nursing management scenarios. Correlations between SJT scores and NUM‐CS total scores were analyzed to evaluate the degree of association between the competency scale and external criterion measures.

All statistical analyses were performed using SPSS Version 26.0 and R Version 4.5.0. SPSS was used for descriptive statistics, item analysis, reliability analysis, and EFA, while R was used for CFA, model comparisons, CR, AVE, and HTMT calculations. AMOS Version 24.0 was used only for drawing structural equation modeling diagrams. Statistical significance was set at a two‐tailed *p* value of < 0.05.

### 2.7. SJT

In addition to the self‐report competency scale, a brief SJT was developed to serve as an external, criterion‐oriented measure of applied NUM competence. The SJT consisted of 12 scenario‐based items reflecting common and high‐stakes nursing management situations in specialized hospital settings, including interdepartmental coordination, staff management, clinical governance, decision‐making under uncertainty, team development, and professional self‐regulation. The scenarios were developed from the qualitative findings, Delphi expert feedback, and typical unit‐level management tasks identified in specialized hospital practice.

Each scenario presented four response options, representing varying levels of managerial effectiveness. Response options were scored based on expert consensus, with higher scores indicating more appropriate managerial judgment. The SJT was designed to assess applied decision‐making rather than latent trait structure and was, therefore, treated as a formative criterion measure rather than a reflective scale.

Accordingly, the SJT items were not subjected to EFA or CFA. Instead, the total SJT score was used to examine criterion‐related validity of the NUM‐CS through correlation with NUM‐CS total scores.

Internal consistency indices such as Cronbach’s alpha were not calculated for the SJT, as the scenarios were designed to represent distinct situational challenges and, therefore, constitute a formative rather than reflective measurement model.

### 2.8. Ethical Considerations

Ethical approval was obtained from the Institutional Review Board of Guangzhou Chest Hospital (Approval No. KY‐2025‐050).

## 3. Results

### 3.1. Sample Characteristics

#### 3.1.1. Specialized Hospital Sample (EFA)

The specialized hospital sample used for EFA comprised 151 participants recruited from 15 hospitals of the same category. Participants were predominantly female, and most were aged between 31 and 50 years. Detailed demographic characteristics of the specialized hospital sample are presented in Table 1.

**TABLE 1:** Demographic characteristics of the specialized hospital sample (EFA, *n* = 151).

Variable	Category	*n*	%
Gender	Male	5	3.3
	Female	146	96.7

Age (years)	≤ 30	10	6.6
	31–40	90	59.6
	41–50	51	33.8

Highest education	Diploma	15	9.9
	Bachelor’s degree	130	86.1
	Master’s degree or above	6	4.0

Professional title	Charge nurse	140	92.7
	Associate chief nurse	9	6.0
	Chief nurse	2	1.3

Years of nursing experience	≤ 10	26	17.2
	11–20	80	53.0
	21–30	37	24.5
	≥ 31	8	5.3

Nurse unit manager reserve candidate	Yes	14	9.3
	No	98	64.9
	Uncertain	39	25.8

#### 3.1.2. Cross‐Setting Hospital Sample (CFA)

The cross‐setting hospital sample used for CFA included 212 participants recruited from 55 hospitals across different categories. The sample was predominantly female, with most participants aged 31–40 years. The majority were recruited from general hospitals, and most institutions were classified as tertiary hospitals. Detailed demographic characteristics of the cross‐setting hospital sample are shown in Table 2.

**TABLE 2:** Demographic characteristics of the cross‐setting hospital sample (CFA, *n* = 212).

Variable	Category	*n*	%
Gender	Male	14	6.6
	Female	198	93.4

Age (years)	≤ 30	10	4.7
	31–40	155	73.1
	41–50	47	22.2

Highest education	Diploma	9	4.2
	Bachelor’s degree	172	81.1
	Master’s degree or above	31	14.6

Professional title	Charge nurse	203	95.8
	Associate chief nurse	7	3.3
	Chief nurse	2	0.9

Years of nursing experience	≤ 10	37	17.5
	11–20	143	67.5
	21–30	27	12.7
	≥ 31	5	2.4

Nurse unit manager reserve candidate	Yes	24	11.3
	No	130	61.3
	Uncertain	58	27.4

Hospital type	Specialized hospital	27	12.7
	General hospital	185	87.3

Hospital level	Tertiary	194	91.5
	Secondary or below	18	8.5

### 3.2. Item Analysis and Internal Consistency

Item analysis and internal consistency reliability were examined using the specialized hospital sample (*n* = 151). The initial version of the NUM‐CS consisted of 35 items.

The overall internal consistency of the NUM‐CS was excellent, with a Cronbach’s α coefficient of 0.979. CITC values ranged from 0.522 to 0.856, exceeding the recommended threshold of 0.40 for all items. Deletion of any individual item did not result in a meaningful increase in Cronbach’s α (range: 0.978–0.979), indicating that all items contributed stably to the overall scale reliability.

These results suggested that the initial 35‐item scale demonstrated strong internal consistency and was suitable for subsequent factor analysis.

### 3.3. EFA

#### 3.3.1. Factorability of the Data

Prior to EFA, the suitability of the data was examined. The KMO measure of sampling adequacy was 0.953, and Bartlett’s test of sphericity was statistically significant (χ^2^ = 5621.024, df = 595, *p* < 0.001), indicating that the correlation matrix was appropriate for factor analysis.

#### 3.3.2. Factor Extraction and Structure

EFA was conducted using principal axis factoring with Promax oblique rotation, given the theoretical expectation that competency dimensions would be correlated. Based on eigenvalues greater than 1.0, inspection of the scree plot, and empirical interpretability, a four‐factor statistical solution emerged.

The four extracted factors jointly explained 73.50% of the total variance, with moderate interfactor correlations (*r* = 0.52–0.75).

Rather than representing an alternative conceptual model, the four‐factor solution was interpreted as reflecting higher‐order empirical clustering of related competencies. This structure was, therefore, treated as an intermediate analytic result, informing subsequent confirmatory testing of the theoretically derived six‐factor model.

#### 3.3.3. Item Retention and Refinement

Item retention was guided by both statistical and theoretical criteria. Items were retained if they demonstrated a primary factor loading ≥ 0.40. In cases of cross‐loading, a minimum difference of 0.20 between the primary and secondary loadings was required, alongside conceptual interpretability.

Two items—“I can implement infection prevention and control requirements” and “When adverse events occur, I can analyze causes and promote improvement in a timely manner”—exhibited insufficient primary factor loadings (0.367 and 0.386, respectively) and were, therefore, removed from the NUM‐CS.

Following item removal, the 33‐item scale was reanalyzed using the same extraction and rotation methods. The revised EFA results remained robust, with a KMO value of 0.953 and a significant Bartlett’s test of sphericity (χ^2^ = 5248.363, df = 528, *p* < 0.001). The four‐factor structure was retained, and the cumulative variance explained increased to 74.28%, indicating improved structural clarity and stability.

The final factor structure and standardized factor loadings are presented in Table 3.

**TABLE 3:** Exploratory factor analysis results for representative items of the NUM‐CS (*n* = 151).

Item	F1	F2	F3	F4
I can integrate training with real clinical projects to enhance team capability	**0.91**			
I can provide timely, specific, and actionable feedback	**0.93**			
I can create psychological safety for staff to speak up and report concerns	**0.89**			
I can role model leadership under high‐pressure situations		**0.78**		
I can make objective decisions based on data and evidence		**0.67**		
I can coordinate solutions when goals across departments conflict			**0.83**	
I can anticipate bottlenecks across pharmacy, laboratory, and imaging interfaces			**0.77**	
I can translate quality management requirements into executable tasks				**0.86**
I can implement nursing policies and guidelines in frontline practice				**0.79**

*Note:* The full factor loading matrix for all 33 items is provided in Supporting Table S1. Bold values indicate the primary factor loadings for each item.

### 3.4. Item Reduction and Final Factor Structure

Item reduction and refinement were conducted using a combination of statistical criteria and theoretical considerations, taking into account the mixed‐format nature of the NUM‐CS. The instrument comprised both self‐report competency statements and scenario‐based situational judgment items designed to assess applied managerial decision‐making.

EFA was performed exclusively on the self‐report items to examine latent competency patterns. Items with low factor loadings, substantial cross‐loadings, or limited conceptual contribution were removed. Although the EFA yielded a four‐factor statistical solution, theoretical mapping indicated that these factors represented higher‐order aggregations of the six competency domains identified in the qualitative phase.

Therefore, the six‐domain competency framework was retained as the final conceptual structure of the self‐report scale. The detailed items and descriptions of the six competency domains (Domain 1–Domain 6) are provided in Supporting Appendix A. This decision was based on the qualitative‐derived theoretical framework, Delphi expert consensus, and subsequent CFA model comparisons rather than on the EFA solution alone. Scenario‐based situational judgment items were treated as a complementary criterion‐oriented component and were not subjected to factor analysis due to their formative nature.

The six‐factor structure of the 33‐item self‐report scale was subsequently evaluated using CFA.

### 3.5. CFA

CFA was conducted using the cross‐setting hospital sample (*n* = 212) to verify the factorial validity of the theoretically proposed six‐factor structure. Maximum likelihood estimation was applied, with each item specified to load on its corresponding latent factor.

To address the discrepancy between the exploratory four‐factor solution and the theoretically derived six‐domain framework, competing measurement models were compared in the independent CFA sample, including a one‐factor model, the EFA‐derived four‐factor model, the theoretically proposed correlated six‐factor model, and a higher‐order six‐factor model. The detailed fit indices are presented in Table 4.

**TABLE 4:** Comparison of alternative measurement models of the NUM‐CS.

Model	*χ* ^2^	df	*χ* ^2^/df	CFI	TLI	RMSEA	90% CI	AIC	BIC
One‐factor model	1911.55	495	3.86	0.834	0.823	0.116	0.111–0.122	2043.55	2265.09
EFA‐derived four‐factor model	1534.27	489	3.14	0.877	0.868	0.101	0.095–0.106	1678.27	1919.94
Correlated six‐factor model	1240.37	480	2.58	0.911	0.902	0.087	0.081–0.093	1402.37	1674.26
Higher‐order six‐factor model	1263.25	489	2.58	0.909	0.902	0.087	0.081–0.093	1407.25	1648.93

Abbreviations: AIC, Akaike information criterion; BIC, Bayesian information criterion; CFI, comparative fit index; RMSEA, root mean square error of approximation; TLI, Tucker–Lewis index.

The one‐factor model showed poor fit, indicating that the NUM‐CS should not be interpreted as a unidimensional measure. The EFA‐derived four‐factor model improved model fit but remained suboptimal. In contrast, the correlated six‐factor model provided the most favorable fit among the tested models, with the highest CFI and the lowest AIC. The higher‐order six‐factor model showed comparable fit and the lowest BIC, suggesting that the six competency domains may also be understood as closely related first‐order dimensions under an overarching NUM competency construct.

In the correlated six‐factor model, all standardized factor loadings were statistically significant (*p* < 0.001) and ranged from 0.687 to 0.932, indicating strong item–factor relationships. CR values ranged from 0.900 to 0.961, and AVE values ranged from 0.680 to 0.802, supporting CR and convergent validity of the six domains. However, interfactor correlations were high (*r* = 0.780–0.967) and HTMT values ranged from 0.805 to 0.971, with several values exceeding the commonly used threshold of 0.90. These findings suggest that the competency domains were conceptually distinguishable but empirically highly interrelated. Therefore, discriminant validity was interpreted cautiously, and the correlated six‐factor model was retained as the primary measurement model, while the higher‐order model provided additional support for interpreting the NUM‐CS as a multidimensional but highly integrated competency measure.

Although the RMSEA value slightly exceeded the conventional 0.08 threshold, the model comparison supported the relative adequacy of the correlated six‐factor model compared with the alternative models. No post hoc error covariances were added, as the purpose of the CFA was to evaluate theoretically specified structures rather than to improve model fit through data‐driven modifications.

### 3.6. Criterion‐Related Validity (SJT)

Criterion‐related validity of the NUM‐CS was examined using a SJT developed in parallel with the self‐report scale. The SJT consisted of 12 scenario‐based items reflecting realistic managerial decision‐making situations in specialized hospital settings. Responses were scored according to expert consensus, with higher scores indicating more appropriate managerial judgment.

Descriptive analysis showed that the 12‐item SJT score ranged from 12 to 24, with a mean score of 22.09 (SD = 2.50), indicating generally high performance in scenario‐based managerial judgment. Pearson correlation analysis showed that total NUM‐CS scores were positively correlated with the 12‐item SJT score (*r* = 0.312, *p* < 0.001, 95% CI: 0.160–0.449). These findings provide preliminary criterion‐related validity evidence and suggest that scenario‐based managerial judgment was related to, but methodologically distinct from, self‐reported competency perceptions.

Taken together, the SJT findings support its use as a complementary external criterion. However, because the SJT was developed within the present study, these findings should be interpreted as preliminary rather than definitive evidence of predictive validity.

## 4. Discussion

### 4.1. Principal Findings

This study developed and psychometrically validated the NUM‐CS using a rigorous multiphase methodological approach, consistent with established guidelines for scale development in nursing and health services research [14, 15]. Drawing on a prior qualitative study and two rounds of Delphi expert consultation, a 33‐item self‐report instrument encompassing six theoretically grounded competency domains was established, aligning with contemporary views that nurse manager competence is multidimensional and context dependent [4, 5].

EFA conducted in a homogeneous specialized hospital sample demonstrated excellent sampling adequacy and a coherent latent structure, providing empirical support for the underlying competency construct. Although a four‐factor statistical solution emerged at the exploratory stage, subsequent CFA in an independent cross‐setting sample supported the theoretically proposed six‐factor model, a pattern commonly observed in leadership and competency research when theory‐driven models are tested following exploratory analyses [16, 17].

The NUM‐CS demonstrated excellent internal consistency across the total scale and all subdomains. The positive correlation between NUM‐CS total scores and the 12‐item SJT score provided preliminary criterion‐related validity evidence, suggesting that self‐reported competency was related to scenario‐based managerial judgment. This finding is consistent with the use of SJTs as scenario‐based measures of applied professional judgment in leadership and healthcare assessment contexts [18, 19]. Overall, these findings provide preliminary support for the reliability and validity of the NUM‐CS across different hospital settings in China, although further research is needed to examine its discriminant validity, predictive validity, and applicability among NUM reserve candidates and practicing NUMs.

### 4.2. Interpretation of the Factor Structure

The factor structure of the NUM‐CS reflects the complex and interrelated nature of NUM competencies in contemporary healthcare settings. Although EFA yielded a four‐factor statistical solution, subsequent model comparison in the independent CFA sample showed that the theoretically proposed correlated six‐factor model provided a more favorable relative fit than the one‐factor and EFA‐derived four‐factor alternatives. This finding suggests that the EFA‐derived four‐factor solution should not be interpreted as a simple replacement of the original six‐domain framework. Instead, the NUM‐CS appears to reflect a hierarchical competency structure, in which six theoretically meaningful domains are closely related to an overarching NUM competency construct [16].

At the exploratory stage, items originally designed to represent six distinct competency domains clustered into four higher‐order empirical factors. This phenomenon suggests that, in practice, multiple managerial competencies are enacted concurrently rather than as discrete skill sets. Similar convergence of leadership, decision‐making, and governance‐related competencies has been reported in empirical studies of nurse managers, particularly in high‐acuity and complex care environments [2, 4].

Importantly, the emergence of a four‐factor solution did not contradict the original theoretical framework but instead reflected broader latent dimensions subsuming conceptually related domains. For example, competencies related to decision‐making, clinical governance, and professional self‐regulation demonstrated substantial overlap at the exploratory level. This finding aligns with prior conceptualizations of the nurse manager role as requiring simultaneous cognitive judgment, ethical accountability, and emotional regulation under conditions of uncertainty and organizational pressure [1, 5].

Likewise, items pertaining to communication, cross‐boundary coordination, and team development clustered together, underscoring the relational and integrative nature of managerial work in complex healthcare systems. This pattern is consistent with relational leadership perspectives and empirical nursing management research highlighting coordination, boundary spanning, and team facilitation as central components of effective nurse manager performance [2].

CFA provided further clarification: the six‐factor model derived from the qualitative phase and Delphi consultation provided the most favorable relative fit among the tested models in an independent, cross‐setting sample. The higher‐order six‐factor model also showed comparable fit, suggesting that the six domains can be understood as closely related first‐order dimensions under an overarching NUM competency construct [17]. The high interfactor correlations, together with several HTMT values exceeding 0.90, indicate limited discriminant separation among some domains and should, therefore, be interpreted as an important psychometric finding. In real‐world nursing management, communication, leadership, clinical governance, decision‐making, team development, and professional self‐regulation are often enacted simultaneously rather than as isolated competencies. For example, managing a high‐risk clinical incident may require cross‐boundary coordination, rapid decision‐making, staff supervision, emotional regulation, and quality improvement actions at the same time. Therefore, the high correlations may reflect the integrated nature of the NUM role. This interpretation is consistent with previous reviews showing that nurse manager competence is multidimensional and context dependent [4, 5]. It is also consistent with empirical evidence linking nurse manager competency to practice environments, missed nursing care, and patient care quality [2].

Taken together, the combined EFA, CFA, model comparison, and higher‐order model findings indicate that the NUM‐CS should be interpreted as a multidimensional but highly integrated competency measure. The six domains are theoretically meaningful and useful for structuring competency assessment and developmental feedback, but they should not be regarded as fully independent constructs. The very high correlations also suggest potential multicollinearity among domain‐level scores, particularly when multiple subdomains are entered simultaneously into predictive models. Domain‐level scores should, therefore, be interpreted cautiously, particularly when the scale is used for reserve candidate comparison, developmental feedback, or competency‐informed selection decisions. Future studies should further examine the dimensionality and discriminant validity of the NUM‐CS using larger samples and alternative modeling approaches, such as bifactor modeling and measurement invariance testing [14, 15].

### 4.3. Validity and Reliability Considerations

The findings provide preliminary support for the reliability and validity of the NUM‐CS, while acknowledging that some aspects of validity require further examination, in line with psychometric standards for health and nursing management instruments [14]. Multiple sources of validity evidence were examined, including internal consistency reliability, construct validity assessed through EFA and CFA, and criterion‐related validity using a SJT.

Internal consistency reliability of the NUM‐CS was excellent. The overall Cronbach’s alpha coefficient exceeded commonly accepted thresholds for applied assessment instruments, and all subscales demonstrated strong internal consistency. CITCs indicated that each item contributed meaningfully to its respective domain, supporting the internal coherence of the scale.

Construct validity was strengthened through a two‐step factor analytic strategy employing independent samples for exploratory and confirmatory analyses. This approach is widely recommended to reduce model overfitting and enhance the generalizability of measurement models [16, 17]. The correlated six‐factor model provided the most favorable relative fit among the tested models, while the higher‐order model supported the interpretation of the NUM‐CS as a multidimensional but highly integrated competency construct.

The positive association between NUM‐CS total scores and the 12‐item SJT score provided preliminary criterion‐related validity evidence. SJTs have been shown to capture applied managerial and leadership competence beyond self‐report measures and are increasingly recommended in healthcare leadership assessment [18, 19]. The observed association suggests that higher perceived competency levels were associated with more appropriate managerial judgment in realistic clinical scenarios.

Taken together, these findings provide preliminary evidence supporting the reliability and several aspects of validity of the NUM‐CS. However, the SJT should be interpreted as a complementary external criterion rather than definitive evidence of predictive validity, and further studies are needed to examine predictive validity using objective organizational and performance outcomes.

### 4.4. Implications for Nursing Management

The findings of this study have important implications for nursing management practice, leadership development, and human resource decision‐making. In the Chinese healthcare context, NUM appointments are often influenced by seniority and administrative considerations, while structured competency‐based assessment remains limited. This misalignment may place newly appointed or prospective NUMs under substantial performance pressure, particularly in specialized and high‐risk clinical settings where managerial decisions have immediate patient safety implications.

In this context, the NUM‐CS offers a structured and empirically grounded tool to support more transparent and competency‐informed identification, development, and formative assessment of NUM reserve candidates, consistent with calls for competency‐based approaches to nursing leadership [2, 5].

The six‐domain structure of the NUM‐CS aligns with contemporary frameworks emphasizing leadership, governance, decision‐making, and professional values as core dimensions of nurse manager performance [4]. Domain‐level feedback may facilitate targeted professional development, succession planning, and competency‐informed developmental feedback, particularly in complex clinical environments.

Furthermore, the use of the SJT as a complementary external criterion provided a supplementary perspective on applied managerial judgment beyond self‐report assessment, which may help reduce reliance on a single self‐report method in leadership evaluation [20]. However, further validation of the SJT is needed before it can be used as a robust external criterion in real‐world nursing management applications.

### 4.5. Limitations and Future Research

First, although participants were recruited from multiple regions and a wide range of hospital types, all data were collected within the Chinese healthcare system. Organizational structures, role expectations, and governance arrangements for NUMs vary substantially across countries, which may limit the direct transferability of the NUM‐CS to other healthcare systems. Future research should examine cross‐cultural applicability through translation, cultural adaptation, and formal tests of measurement invariance across different national and organizational contexts.

Second, the psychometric evaluation was based on cross‐sectional data. While internal consistency, factorial validity, composite reliability, convergent validity, and preliminary criterion–related validity were supported, longitudinal evidence is needed to assess test–retest reliability and sensitivity to change. Such evidence would be particularly important for evaluating the utility of the NUM‐CS in leadership development programs, succession planning, and competency‐based interventions over time.

Third, criterion‐related validity was examined using a SJT focused on applied managerial decision‐making. Although this approach strengthened validity evidence by incorporating a scenario‐based external criterion, the study did not include longitudinal evidence on appointment readiness, role transition, or managerial performance after appointment. The 12‐item SJT score was positively correlated with NUM‐CS total scores, supporting its role as a complementary external criterion. However, because the SJT was developed within the present study, the criterion‐related validity evidence should be regarded as preliminary. Future studies should examine whether NUM‐CS scores predict subsequent selection, role transition, and managerial performance among NUM reserve candidates.

Fourth, another limitation concerns the discriminant validity of the NUM‐CS subdomains. Although CR and AVE supported composite reliability and convergent validity, the high interfactor correlations and several HTMT values exceeding 0.90 indicated limited discriminant separation among some competency domains. This suggests that the six domains should not be interpreted as fully independent constructs. Instead, they may represent closely related facets of an overarching NUM competency construct, which is consistent with the acceptable fit of the higher‐order model. Therefore, domain‐level scores should be interpreted cautiously, particularly when the NUM‐CS is used for reserve candidate comparison, developmental feedback, or competency‐informed selection decisions. Future studies with larger and more diverse samples should further examine the dimensionality and discriminant validity of the NUM‐CS using additional approaches, such as bifactor modeling, measurement invariance testing, and predictive validity assessment.

Fifth, the use of self‐report measures may have introduced social desirability bias, particularly in hierarchical organizational cultures. Future studies could incorporate supervisor ratings, peer assessments, objective performance indicators, or multisource feedback to provide more comprehensive validity evidence.

Finally, although the item pool and competency framework were derived from qualitative data involving NUMs and nursing management stakeholders, the large‐sample psychometric validation focused on nurses eligible for future NUM appointments rather than currently practicing NUMs. This sampling strategy was consistent with the intended use of the NUM‐CS for identifying, developing, and informing the selection of NUM reserve candidates. Nevertheless, future longitudinal studies should examine whether NUM‐CS scores predict subsequent appointment, role transition, and managerial performance after appointment.

Despite these limitations, the present study provides a preliminarily validated, practice‐oriented competency assessment tool for identifying NUM reserve candidates, supporting leadership development, and informing succession planning. Future research building on this work may further refine the NUM‐CS and extend its application in nursing management research, leadership development, and workforce planning.

## 5. Conclusion

This study developed and preliminarily validated the NUM‐CS, a practice‐oriented instrument grounded in empirical evidence and nursing management theory. The scale demonstrated strong internal consistency and preliminary support for factorial and criterion‐related validity across diverse hospital settings. The use of an external SJT provided supplementary evidence that NUM‐CS scores were related to applied managerial judgment. The instrument provides a practical foundation for competency‐based identification, development, and formative assessment of NUM reserve candidates, particularly in specialized and high‐risk clinical environments. Further longitudinal validation is needed to examine its predictive value for subsequent appointment, role transition, and managerial performance.

## Author Contributions

Conceptualization: Minjuan Zhou and Jianmei Chen. Methodology: Jianmei Chen. Formal analysis: Jianmei Chen and Shuxia Xie. Investigation: Jianmei Chen, Shuxia Xie, Jianwen Li, Nianhao Zhong, Xiaoyan Tan, Haixin Jiang, and Zhengwei Lan. Writing–original draft: Jianmei Chen. Writing–review and editing: Minjuan Zhou. Supervision: Minjuan Zhou.

## Funding

This research received no specific grant from funding agencies in the public, commercial, or not‐for‐profit sectors.

## Disclosure

All authors have read and approved the final manuscript.

## Conflicts of Interest

The authors declare no conflicts of interest.

## Supporting Information

Additional supporting information can be found online in the Supporting Information section.

## Supporting information


**Supporting Information 1** Supporting Table S1 presents the complete factor loading matrix of the final 33‐item NUM‐CS derived from exploratory factor analysis. Supporting Appendix A provides the situational judgment test (SJT) scenarios and scoring information developed for assessing Nurse Unit Manager competencies, including the six competency domains (Domain 1–Domain 6).

**Supporting Information 2** The completed STROBE checklist is provided as supporting information to demonstrate adherence to reporting recommendations for observational studies.

## Data Availability

The datasets generated and analyzed during the current study are available from the corresponding author upon reasonable request. No other publications or manuscripts using these data are currently under consideration or submitted elsewhere.

## References

[1] Agency for Healthcare Research and Quality , The Role of the Nurse Manager, 2018, U.S. Department of Health & Human Services, https://www.ahrq.gov/hai/cusp/modules/nursing/index.html.

[2] Warshawsky N. E. , Cramer E. , Grandfield E. M. , and Schlotzhauer A. E. , The Influence of Nurse Manager Competency on Practice Environment, Missed Nursing Care, and Patient Care Quality, Journal of Nursing Management. (2022) 30, no. 6, 1981–1989, 10.1111/jonm.13649. 35474621

[3] Billiau L. , Taghon D. , Duprez V. , Eeckloo K. , and Malfait S. , Instruments for Measuring Head Nurses’ Competencies in a Hospital Setting: a Scoping Review, BMC Nursing. (2025) 24, no. 1, 10.1186/s12912-025-03719-0. PMC12351982 40814107

[4] Filomeno L. , Forte D. , Di Simone E. et al., Systematic Review and Psychometric Properties Analysis of first-middle-and top-level Nurse Manager’s Core Competencies Instruments, Journal of Nursing Management. (2024) 2024, 1–15, 10.1155/2024/2655382. PMC11918900 40224784

[5] González-García A. , Pinto-Carral A. , Pérez-González S. , and Marqués-Sánchez P. , Nurse Managers’ Competencies: a Scoping Review, Journal of Nursing Management. (2021) 29, no. 6, 1410–1419, 10.1111/jonm.13380. 34018273

[6] Pabico C. , Warshawsky N. E. , and Park S. H. , Psychometric Properties of the Nurse Manager Competency Instrument for Research, Journal of Nursing Measurement. (2023) 31, no. 2, 273–283, 10.1891/JNM-2021-0040. 37277154

[7] Ivziku D. , Filomeno L. , Forte D. et al., Reliability and Validity of the Italian Version of the Chase Nurse Manager Competencies Scale, International Journal of Nursing Sciences. (2024) 11, no. 3, 338–348, 10.1016/j.ijnss.2024.06.001. 39156677 PMC11329004

[8] Wang J. , Yang L. , Yu J. , Wu C. , and Yang H. , A Systematic Review of Evaluation Tools for Core Competencies of Head Nurses Based on COSMIN Guidelines, Chinese Journal of Nursing Education. (2023) 20, no. 11, 1347–1354, 10.3761/j.issn.1672-9234.2023.11.011.

[9] McClelland D. C. , Testing for Competence Rather than for Intelligence, American Psychologist. (1973) 28, no. 1, 1–14, 10.1037/h0034092. 4684069

[10] Boyatzis R. E. , The Competent Manager: A Model for Effective Performance, 1982, John Wiley & Sons.

[11] American Organization for Nursing Leadership , AONL Nurse Manager Competencies, American Organization for Nursing Leadership. (2015) https://www.aonl.org/nurse-manager-competencies.

[12] Lee J. and Kim M. , Building Managerial Competence in Nurse Unit Managers: Insights from a Qualitative Study, BMC Nursing. (2026) 25, no. 1, 10.1186/s12912-026-04695-9. PMC13261823 42046120

[13] Scally G. and Donaldson L. J. , Clinical Governance and the Drive for Quality Improvement in the New NHS in England, BMJ. (1998) 317, no. 7150, 61–65, 10.1136/bmj.317.7150.61. 9651278 PMC1113460

[14] DeVellis R. F. , Scale Development: Theory and Applications, 2017, 4th edition, Sage Publications.

[15] Boateng G. O. , Neilands T. B. , Frongillo E. A. , Melgar-Quiñonez H. R. , and Young S. L. , Best Practices for Developing and Validating Scales for Health, Social, and Behavioral Research: a Primer, Frontiers in Public Health. (2018) 6, 10.3389/fpubh.2018.00149. PMC6004510 29942800

[16] Brown T. A. , Confirmatory Factor Analysis for Applied Research, 2015, 2nd edition, Guilford Press.

[17] Kline R. B. , Principles and Practice of Structural Equation Modeling, 2016, 4th edition, Guilford Press.

[18] Lievens F. , Peeters H. , and Schollaert E. , Situational Judgment Tests: a Review of Recent Research, Personnel Psychology. (2008) 61, no. 2, 287–317, 10.1111/j.1744-6570.2008.00119.x.

[19] Patterson F. , Zibarras L. D. , and Ashworth V. , Situational Judgement Tests in Medical Education and Training: Research, Theory and Practice: AMEE Guide No. 100, Medical Teacher. (2016) 38, no. 1, 3–17, 10.3109/0142159X.2015.1072619. 26313700

[20] Podsakoff P. M. , MacKenzie S. B. , Lee J. Y. , and Podsakoff N. P. , Common Method Biases in Behavioral Research: a Critical Review of the Literature and Recommended Remedies, Journal of Applied Psychology. (2003) 88, no. 5, 879–903, 10.1037/0021-9010.88.5.879. 14516251

