# Supplementary material for: Development and Validation of the Nurse Unit Manager Competency Scale (NUM‐CS): A Multicenter Study
Source: J Nurs Manag. 2026 Aug 4;2026:9388493. doi: 10.1155/jonm/9388493 (PMC13438863; doi:10.1155/jonm/9388493)
Supplement: Supplementary file 1 [file JONM-2026-9388493-s001.docx]

**Table S1. Standardised factor loadings of the final 33-item Nurse Unit Manager Competency Scale (NUM-CS) after Promax rotation (n = 151)**

| **Item** | **Factor 1** | **Factor 2** | **Factor 3** | **Factor 4** |
| --- | --- | --- | --- | --- |
| I can integrate training with real clinical projects to enhance team capability | 0.91 |  |  |  |
| I can provide timely, specific, and actionable feedback | 0.93 |  |  |  |
| I can create psychological safety for staff to speak up and report concerns | 0.89 |  |  |  |
| I can arrange developmentally appropriate challenge tasks for staff and provide support | 0.78 |  |  |  |
| I can conduct regular coaching or debriefing to promote reflective learning | 0.73 |  |  |  |
| I can identify and support staff with development potential | 0.72 |  |  |  |
| I can balance patient safety and team efficiency when resources are constrained | 0.78 |  |  |  |
| I can make rapid judgments and take action when situations are urgent or information is incomplete | 0.59 |  |  |  |
| I can synthesize information from multiple sources before making decisions | 0.76 |  |  |  |
| I can initiate risk reporting procedures at critical moments | 0.76 |  |  |  |
| I can clearly document decision rationales and follow-up actions | 0.66 |  |  |  |
| I can use structured decision-making or analytical tools (e.g., 5 Whys, fishbone diagrams) | 0.77 |  |  |  |
| I can remain emotionally stable and professional under high-pressure situations | 0.63 |  |  |  |
| I can uphold principles and treat all staff equitably |  | 0.61 |  |  |
| I can demonstrate leadership by example during high-stress situations |  | 0.78 |  |  |
| I can handle interpersonal conflicts within the team fairly and promptly |  | 0.56 |  |  |
| I can clearly define expectations, roles, and boundaries when delegating tasks |  | 0.86 |  |  |
| I can make objective decisions based on data and evidence |  | 0.67 |  |  |
| I can explain organizational changes and address staff concerns |  | 0.47 |  |  |
| I can uphold fairness and avoid bias in management decisions |  | 0.75 |  |  |
| I can coordinate solutions when goals across departments conflict |  |  | 0.83 |  |
| I can anticipate bottlenecks across pharmacy, laboratory, and imaging interfaces |  |  | 0.77 |  |
| I can maintain smooth interdepartmental collaboration during peak or emergency situations |  |  | 0.75 |  |
| I can establish stable communication channels with functional departments to improve workflows |  |  | 0.80 |  |
| I can ensure accurate handovers and escalation following standardized procedures |  |  | 0.85 |  |
| I can translate upper-level decisions into routine operational practice |  |  | 0.54 |  |
| I can identify potential clinical risks and intervene proactively |  | 0.54 |  |  |
| I can organize brief trainings or drills to improve procedural compliance |  | 0.55 |  |  |
| I can promote the implementation of nursing policies and clinical guidelines |  |  |  | 0.62 |
| I can translate quality management requirements into executable tasks |  |  |  | 0.64 |
| I can convey patient safety–centered values to the team | 0.53 |  |  |  |
| I can maintain continuous learning and reflection amid heavy workloads | 0.57 |  |  |  |
| I can ensure equitable access to learning and development opportunities for staff | 0.48 |  |  |  |

Extraction method: Principal axis factoring

Rotation method: Promax (κ = 4)

KMO = 0.953; Bartlett’s test of sphericity χ² = 5248.363, p < 0.001

The four-factor solution explained 74.28% of the total variance.

Note. Only the primary factor loading for each retained item is displayed.

**Supplementary Appendix A**

Situational Judgment Test (SJT) for Nurse Unit Manager Competencies

Instructions to participants

The following scenarios describe realistic nursing management situations.

For each scenario, please select the option that best reflects the action you would be most likely to take.

**Domain 1: Communication and Cross-boundary Coordination (C)**

Scenario C6

One afternoon, a dispute arises between your unit and the imaging department regarding patient queue priority. The imaging staff accuse nurses from your unit of “jumping the queue.” When you arrive, emotions on both sides are escalating.

What would you most likely do?

A. Ask both sides to step back and wait for the nurse manager to resolve the issue

B. First comfort your own staff and then see how the situation develops

C. Immediately clarify the key concerns of both sides, address misunderstandings, and facilitate a mutually agreed solution

D. Ask both sides to calm down and return to their departments before reporting the issue

Scenario C8

The pharmacy suddenly changes its medication pickup process, causing delays and misunderstandings with the ward staff.

What would you most likely do?

A. Wait for the pharmacy to provide an explanation

B. Ask ward staff to find their own way to adapt

C. Proactively contact the pharmacy to clarify the process change and communicate contingency plans to ward staff

D. Wait for instructions from the nurse manager

**Domain 2: Leadership and Personnel Management (L)**

Scenario L6

A senior nurse consistently delays documentation, but she is well-liked by colleagues, and no one wants to point out the problem.

What would you most likely do?

A. Ignore the issue to avoid conflict

B. Gently remind her to pay attention

C. Have a private discussion to explain the impact of the behaviour and jointly identify improvement strategies

D. Refer the issue directly to the nurse manager

Scenario L8

Your unit is implementing a new workflow, but information is incomplete and the situation is complex. Staff are waiting for your guidance.

What would you most likely do?

A. Wait for detailed instructions from the nurse manager

B. Follow previous practices as a rough guide

C. Develop a feasible plan based on available information and adjust it during implementation

D. Avoid making a decision and let others act first

**Domain 3: Clinical Governance and Risk Management (G)**

Scenario G6

During a night shift, a critically ill patient is admitted, and medical orders are frequently updated. A novice nurse appears anxious.

What would you most likely do?

A. Ask the novice nurse to try following the protocol independently

B. Rely on your own experience and carry out the orders yourself

C. Guide the novice nurse to review each order step by step and implement care according to protocol

D. Let the novice nurse complete the tasks and review them afterward

Scenario G8

The hospital reports three recent catheter dislodgement incidents and requires units to conduct a review.

What would you most likely do?

A. Conduct a minimal, formal review

B. Briefly discuss two or three cases

C. Organise a team discussion to identify high-risk steps and develop improvement actions

D. Observe how other units respond before taking action

**Domain 4: Decision-making and Problem-solving (D)**

Scenario D5

Thirty minutes before the end of a night shift, a patient with a complex condition is admitted, but information is incomplete.

What would you most likely do?

A. Wait until the physician provides complete information

B. Provide minimal care and leave the rest to the next shift

C. Rapidly identify key issues and develop a temporary management plan

D. Leave the situation entirely for the next shift

Scenario D8

During a shift change, two patients require urgent attention at the same time. One patient has sudden shortness of breath, while another patient’s family member is repeatedly requesting discharge-related explanations. The available nursing staff are limited.

What would you most likely do?

A. Deal with the family member first to avoid complaints

B. Ask staff to manage the situation themselves while you continue routine work

C. Rapidly assess the urgency of both situations, prioritise the patient with potential clinical deterioration, assign staff clearly, and arrange follow-up communication with the family member

D. Wait for the nurse manager or physician to decide the order of priority

**Domain 5: Team Development and Talent Cultivation (T)**

Scenario T5

A task continues to go wrong, and team members suggest that you “just do it yourself.”

What would you most likely do?

A. Do it yourself to save time

B. Demonstrate the task once for the team

C. Break the task down, assign it to team members, and coach them through completion

D. Refer the task to the nurse manager

Scenario T8

A junior nurse has repeatedly shown potential in clinical work but lacks confidence when taking responsibility for more complex tasks. The team is busy, and some colleagues think it is faster to assign tasks only to experienced nurses.

What would you most likely do?

A. Avoid assigning challenging tasks to the junior nurse

B. Assign the task directly without guidance to test her ability

C. Provide a developmentally appropriate task, offer clear guidance, and give timely feedback after completion

D. Ask the nurse manager to decide whether the junior nurse should be trained

**Domain 6: Professional Self-regulation and Values (S)**

Scenario S6

The ward is extremely busy, and you are involved in an emergency rescue. Another patient requires strict adherence to standard procedures.

What would you most likely do?

A. Simplify the procedure

B. Complete part of the procedure and make up the rest later

C. Strictly follow institutional protocols despite workload pressure

D. Ask someone else to handle it and move on

Scenario S8

After three consecutive night shifts, you feel emotionally exhausted. A family member expresses dissatisfaction with nursing arrangements.

What would you most likely do?

A. Respond emotionally and argue back

B. Ask the family member to speak to the nurse manager

C. Control your emotions and calmly explain the nursing arrangements

D. Ignore the complaint and continue your work

**Scoring**

Response options were scored based on expert consensus, with higher scores indicating responses more consistent with effective nursing management practice. Each item was scored from 1 to 2 according to expert-rated appropriateness, yielding a total possible score range of 12 to 24 for the 12-item SJT. The SJT score was used only as an external criterion measure and was not included in the NUM-CS self-report scale score.
