# Supplementary material for: Development and Validation of the Nurse Unit Manager Competency Scale (NUM‐CS): A Multicenter Study
Source: J Nurs Manag. 2026 Aug 4;2026:9388493. doi: 10.1155/jonm/9388493 (PMC13438863; doi:10.1155/jonm/9388493)
Supplement: Supplementary file 2 [file JONM-2026-9388493-s002.docx]

**STROBE Checklist**

**Manuscript: Development and Validation of the Nurse Unit Manager Competency Scale (NUM-CS): A Multicentre Study**

**Reporting guideline used:** STROBE Statement checklist for observational studies, completed for a cross-sectional methodological scale-development and validation study.

| **Section** | **Item No.** | **STROBE recommendation** | **Reported location in manuscript** | **How this item is addressed** |
| --- | --- | --- | --- | --- |
| Title and abstract | 1 | Indicate the study design with a commonly used term in the title or abstract; provide an informative and balanced summary of what was done and what was found. | Title; Abstract—Aim, Background, Methods, Results, Conclusion | The title identifies the study as a multicentre scale development and validation study. The structured abstract summarises the design, samples, psychometric analyses, and key findings. |
| Introduction: Background/rationale | 2 | Explain the scientific background and rationale for the investigation being reported. | 1. Introduction | The Introduction describes the importance of Nurse Unit Managers, the need for competency-based assessment, limitations of existing tools, and the specialised/high-risk Chinese hospital context. |
| Introduction: Objectives | 3 | State specific objectives, including any prespecified hypotheses. | End of 1. Introduction | The study aim is stated: to develop and preliminarily validate a competency-based assessment instrument for identifying and developing Nurse Unit Manager reserve candidates across hospital settings in China. |
| Methods: Study design | 4 | Present key elements of study design early in the paper. | 2.1 Study design and overview | The manuscript identifies a multi-phase methodological study including item generation, Delphi consultation, pilot testing, EFA, CFA, reliability testing, and criterion-related validity assessment. |
| Methods: Setting | 5 | Describe the setting, locations, and relevant dates, including periods of recruitment, exposure, follow-up, and data collection. | 2.3 Content validity; 2.4 Participants and sampling; 2.5 Data collection procedures | The Delphi period, recruitment settings, hospital types, and data collection period from May to December 2025 are reported. |
| Methods: Participants | 6 | Give the eligibility criteria and sources and methods of selection of participants. | 2.4 Participants and sampling | The specialised hospital EFA sample and cross-setting CFA sample are described, with inclusion criteria, exclusion of current Nurse Unit Managers, and sampling sources. |
| Methods: Variables | 7 | Clearly define all outcomes, exposures, predictors, potential confounders, and effect modifiers; give diagnostic criteria where applicable. | 2.2 Item generation; 2.6 Psychometric evaluation; 2.7 Situational Judgment Test | The primary variables are NUM-CS self-report item scores, subscale/total scores, SJT scores, and psychometric indicators including reliability, factor structure, CR, AVE, HTMT, and criterion-related validity. |
| Methods: Data sources/measurement | 8 | For each variable of interest, give sources of data and details of methods of assessment or measurement; describe comparability of assessment methods if more than one group. | 2.5 Data collection procedures; 2.6 Psychometric evaluation; 2.7 Situational Judgment Test | Data were collected using Wenjuanxing. Measurement of self-report competency items, SJT scoring, EFA/CFA procedures, and reliability/validity indices are described. |
| Methods: Bias | 9 | Describe any efforts to address potential sources of bias. | 2.5 Data collection procedures; 4.5 Limitations and future research | The manuscript describes anonymous online completion and validity screening. Limitations address self-report/social desirability bias, cross-sectional design, and the need for future multisource validation. |
| Methods: Study size | 10 | Explain how the study size was arrived at. | 2.4 Participants and sampling; 3.1 Sample characteristics | The manuscript reports two independent samples: n = 151 for EFA and n = 212 for CFA. The sampling strategy and use of independent samples for different psychometric stages are described. |
| Methods: Quantitative variables | 11 | Explain how quantitative variables were handled in the analyses; describe groupings if applicable. | 2.2 Item generation; 2.6 Psychometric evaluation; 2.7 Situational Judgment Test | Likert scoring, total/subscale scores, SJT scoring, and continuous psychometric indicators are described. Demographic variables are summarised categorically in Tables 1 and 2. |
| Methods: Statistical methods | 12 | Describe all statistical methods, including those used to control for confounding; describe methods used to examine subgroups/interactions, missing data, sensitivity analyses, and software as applicable. | 2.6.1–2.6.5 Psychometric evaluation | The manuscript reports descriptive statistics, item analysis, Cronbach’s alpha, EFA, CFA, model comparisons, CR, AVE, HTMT, criterion-related correlation analysis, software versions, and significance threshold. |
| Results: Participants | 13 | Report numbers of individuals at each stage of study; give reasons for non-participation at each stage where applicable; consider a flow diagram. | 3.1 Sample characteristics; Tables 1 and 2 | The final EFA and CFA sample sizes are reported, with demographic characteristics presented separately for the specialised and cross-setting samples. A flow diagram was not used because this was an online methodological survey without longitudinal follow-up. |
| Results: Descriptive data | 14 | Give characteristics of study participants and information on exposures and potential confounders; indicate number of participants with missing data for each variable if relevant. | 3.1 Sample characteristics; Tables 1 and 2 | Participant characteristics are reported by gender, age, education, professional title, nursing experience, reserve candidate status, hospital type, and hospital level. |
| Results: Outcome data | 15 | Report numbers of outcome events or summary measures. | 3.2–3.6 Results; Tables 3 and 4; Supplementary Table S1 | Psychometric outcomes are reported, including reliability, factorability, EFA variance explained, factor loadings, CFA fit indices, CR, AVE, HTMT, and NUM-CS/SJT correlation. |
| Results: Main results | 16 | Give unadjusted estimates and, if applicable, confounder-adjusted estimates and their precision; make clear which confounders were adjusted for and why. | 3.2–3.6 Results | Main psychometric results are reported with relevant statistics, including Cronbach’s alpha, KMO, Bartlett’s test, factor loadings, model fit indices, CR, AVE, HTMT, and correlation coefficients with confidence intervals. |
| Results: Other analyses | 17 | Report other analyses done, such as analyses of subgroups/interactions and sensitivity analyses. | 3.5 Confirmatory factor analysis; Table 4 | Competing measurement model analyses are reported, including one-factor, EFA-derived four-factor, correlated six-factor, and higher-order six-factor models. |
| Discussion: Key results | 18 | Summarise key results with reference to study objectives. | 4.1 Principal findings | The Discussion summarises the development of a 33-item NUM-CS, evidence from EFA/CFA, internal consistency, and preliminary criterion-related validity with the SJT. |
| Discussion: Limitations | 19 | Discuss limitations of the study, taking into account sources of potential bias or imprecision; discuss direction and magnitude of potential bias. | 4.5 Limitations and future research | Limitations include Chinese healthcare context, cross-sectional design, preliminary SJT criterion evidence, discriminant validity concerns, self-report bias, and absence of current Nurse Unit Managers in the large-sample validation. |
| Discussion: Interpretation | 20 | Give a cautious overall interpretation of results considering objectives, limitations, multiplicity of analyses, results from similar studies, and other relevant evidence. | 4.1–4.4 Discussion | The manuscript interprets the NUM-CS as a multidimensional but highly integrated competency measure and discusses theoretical and practical implications while avoiding overstatement. |
| Discussion: Generalisability | 21 | Discuss the generalisability or external validity of study results. | 4.5 Limitations and future research | Generalisability is discussed with respect to the Chinese healthcare system, specialised/high-risk settings, cross-cultural applicability, and future measurement invariance testing. |
| Other information: Funding | 22 | Give the source of funding and the role of funders for the present study and, if applicable, for the original study on which the present article is based. | 6.2 Funding; 6.1 Declaration of Competing Interest | The manuscript states that the research received no specific grant and includes a declaration of competing interest. |
